# Older Age and Abnormal Pulmonary Ventilation Function Do Not Increase the Risk of Pulmonary Hemorrhage Caused by CT-Guided Percutaneous Core Needle Biopsy

**DOI:** 10.1155/2022/5238177

**Published:** 2022-08-05

**Authors:** Xuejuan Yu, Chunhai Li, Dexiang Wang, Bo Liu, Haipeng Jia, Wei Zhou

**Affiliations:** ^1^Department of Radiation Oncology, Qilu Hospital, Cheeloo College of Medicine, Shandong University, 107 Wenhuaxi Road, Jinan 250012, Shandong, China; ^2^Department of Radiology, Qilu Hospital, Cheeloo College of Medicine, Shandong University, 107 Wenhuaxi Road, Jinan 250012, Shandong, China; ^3^Department of Respiratory Medicine, Qilu Hospital, Cheeloo College of Medicine, Shandong University, 107 Wenhuaxi Road, Jinan 250012, Shandong, China

## Abstract

**Purpose:**

The aim of this study was to analyze the differences in risk factors for pulmonary hemorrhage in elderly and young patients with percutaneous computed tomography-guided needle biopsies (PCNBs). The correlations between the incidence of pulmonary hemorrhage and pulmonary function indicators before CT-guided PCNB were also discussed.

**Methods:**

Between January 2018 and December 2019, 1,100 consecutive patients underwent CT-guided PCNBs at Qilu Hospital. Both univariate and multivariate logistic regression analyses identified risk factors for hemorrhage.

**Results:**

The occurrence of pulmonary hemorrhage was 22.1% in elderly patients and was 22.6% in young patients. In elderly patients, pulmonary hemorrhage was significantly influenced by needle depth to the lesion and dwell time, while in young patients, pulmonary hemorrhage was independently associated with lesion size, needle depth to the lesion, and dwell time. However, pulmonary function parameters, including FVC (% pred), FEV_1_ (% pred), FEV_1_/FVC ratio (%), small airway function parameters (FEF_50%_, FEF_75%_, and FEF_25–75%_), and large airway function parameters (MVV, PEF, and FEF_25%_), were not risk factors for hemorrhage. Furthermore, the incidence of pulmonary hemorrhage was not associated with different types of pulmonary dysfunctions. The risk of pulmonary hemorrhage did not increase with the severity of pulmonary dysfunctions.

**Conclusions:**

In this study, age is no longer a risk factor in evaluating pulmonary hemorrhage. Longer needle depth to the lesion and longer dwell time were significantly high risk factors of hemorrhage in both elderly patients and young patients. Patients with severe pulmonary dysfunctions did not show increased risks of pulmonary hemorrhage here.

## 1. Introduction

Percutaneous computed tomography-guided needle biopsies (PCNBs) have been favored for the diagnosis of lung lesions in clinic because of accurate diagnostic rate up to 90% and minimal invasiveness [1]. Recent advances in immunotherapy and targeted therapy also required clinical specimens for molecular test. However, fine-needle (usually 22-gauge) aspiration (FNA) specimens were found to be insufficient to perform DNA analysis. But PCNBs with 18–20-gauge needles were associated with high rates of complications, most of which were mild. Both pulmonary hemorrhage and pneumothorax were the most common complications after PCNBs, while tumor implantation and air embolism were relatively rare [2]. The incidence of pneumothorax was reported to be approximately 20%–27%, the incidence of pulmonary hemorrhage ranged from 5% to 11%, and hemoptysis was 7%, all of which were not so severe that surgery treatment was received [1, 3]. Systemic air embolism, with an incidence range from 0.02% to 0.07%, could be fatal [3]. Although the incidence of pulmonary hemorrhage complications was not as high as that of pneumothorax, the consequence of pulmonary hemorrhage was potentially more serious. Sometimes, severe pulmonary hemorrhage could even be life-threatening. An interventional radiologist was more worried about the occurrence of massive bleeding than pneumothorax. It is known that certain lesion and technical characteristics have influenced the incidence of these complications. For example, needle depth to the lesion longer than 2.0 cm is a strong predictor for pulmonary hemorrhage. For subpleural lesions ≤2.0 cm in depth, a higher needle-pleura angle may reduce the risk of pulmonary hemorrhage [4]. Interestingly, An et al. [5] reported that post-biopsy hemorrhage may be a protective factor for pneumothorax. In addition, recent studies reported that application of 3-dimensionally printed coplanar template improved diagnostic yield of CT-guided PCNBs for pulmonary nodules, especially for pulmonary lesions ≤2.0 cm [6]. For individuals with clinically suspicious lung lesions that initially received negative CT-guided PCNB findings, 1.0-T open MR-guided secondary lung biopsy was a safe and effective secondary diagnostic approach [7].

Nowadays, with the aging of the population, more and more elderly patients undergo pulmonary puncture, most of whom have abnormal pulmonary ventilation function. In the studied 1,100 consecutive patients, who received PCNBs at our hospital from January 2018 to December 2019, 39.5% of patients were over 65 years old. The aim of this study was to evaluate the possible risk factors of pulmonary hemorrhage, including patient demographics, target lesions, biopsy procedures, and histopathological diagnosis. In particular, we sought to analyze the differences in risk factors for pulmonary hemorrhage in elderly and young patients with PCNBs. Furthermore, we discussed how to predict this complication by pulmonary function indicators. We further assessed the correlation between the incidence of pulmonary hemorrhage and pulmonary dysfunctions before CT-guided PCNB.

## 2. Materials and Methods

### 2.1. Patients

Between January 2018 and December 2019, 1,100 consecutive patients underwent CT-guided PCNBs at Qilu Hospital. There were 648 men and 452 women (median age: 62.0 years; range: 18–98 years). Of these patients, 343 received pulmonary function test within one week before PCNB. The study was approved by the Medical Ethics Committee of Qilu Hospital of Shandong University (registration number: KYLL-202008-145) with a waiver of informed consent. All data were anonymized and recorded including characteristics of the patient demographics, target lesions, biopsy procedures, and pathological diagnosis as shown in Table 1. Lesions in the middle, lingular, and lower lobes were identified as the lower lesion sites. The needle-pleural angle was determined as the smallest angle at the puncture point between the needle and the tangent line of the pleura [8]. Complications such as pulmonary hemorrhage were recorded. Pulmonary hemorrhage was defined as ground-glass opacification or new consolidation surrounding the puncture needle track on post-biopsy CT scans [9]. The severity of hemorrhage was divided into the following: grade 0, no pulmonary hemorrhage; grade 1 (mild), needle tract bleeding width ≤2 cm; grade 2 (severe), bleeding width >2 cm, segmental or lobar hemorrhage, or hemothorax [10].

### 2.2. Biopsy Procedures

**Figure 1:**
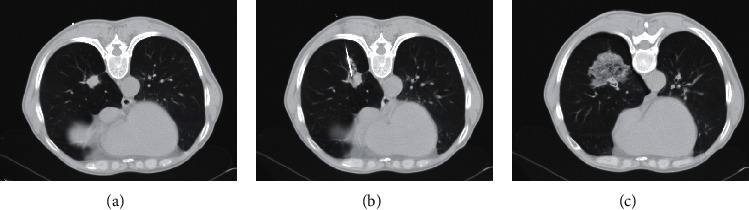
Pulmonary hemorrhage after CT-guided core needle biopsy.

Patients were routinely given blood pressure measurement before and after the procedures. All biopsies were taken under CT guidance by one experienced intervention team. Bleeding profiles, including platelet count, prothrombin (PT), and activated partial thromboplastin time (APTT), were monitored before the procedure. Anticoagulant or antithrombotic drugs, such as warfarin, aspirin, or low molecular weight heparin, were withheld for one week before the biopsy. Almost all patients underwent contrast-enhanced chest CT scans to avoid larger vessels before biopsies. Informed consent was obtained from all cases. The biopsies were taken in a stable and comfortable position such as supine, prone, or lateral decubitus position, taking into account the shortest path of the target lesion and avoiding fissures, bullae or visible vessels. Local anesthesia was given with 1% lidocaine, and the biopsies were implemented with a coaxial cutting needle system (BioPince, Argon Medical Devices, Frisco, Texas; 17-gauge outer sheath; 18-gauge cutting needle). Most CT scans were performed at the end of an exhalation, followed by a small inhalation and holding the breath. Patients with full expiration and full inspiration were not able to easily hold their breath for a relatively long time, especially for patients with poor lung function. Complications such as pneumothorax and pulmonary hemorrhage were evaluated by post-biopsy CT scanning (Figure 1). After biopsies, patients were fasted and observed routinely for 4 hours. Patients with severe complications were hospitalized until their conditions were relieved.

### 2.3. Pulmonary Function Test

Out of the 1,100 biopsy patients retrospectively analyzed, 343 patients underwent pulmonary function tests (simple spirometries) in Qilu Hospital within one week prior to PCNBs. Obstructive dysfunction was characterized by a normal forced vital capacity (FVC) and a reduction in forced expiratory volume in one second/FVC ratio (FEV_1_/FVC < 70%) [11]. FEV_1_ percent predicted (FEV_1_% pred) was not considered in the diagnosis of obstructive dysfunction. Restrictive dysfunction was deﬁned as a normal FEV_1_/FVC ratio and a reduction in FVC [12]. A mixed pulmonary dysfunction was determined as obstruction and restriction existing simultaneously [11, 13]. Furthermore, the severity of obstructive, restrictive, and mixed pulmonary dysfunctions was classified into five levels according to the FEV_1_% pred (mild, FEV_1_% pred ≥ 70; moderate, 60 ≤ FEV_1_% pred < 70; moderately severe, 50 ≤ FEV_1_% pred < 60; severe, 35 ≤ FEV_1_% pred < 50; very severe, FEV_1_% pred < 35) [12]. In addition, small airway dysfunction was determined to be that two of the three parameters FEF_50%_, FEF_75%_, and FEF_25–75%_ were below 65% of the predicted value, provided that the normal ventilation function parameters such as FEV_1_, FEV_1_/FVC ratio, and FVC were normal.

### 2.4. Statistical Analysis

Parametric variables with non-normal distribution were summarized as median with lower quartile to upper quartile and nonparametric variables as numbers (percentage). Between two groups with or without pulmonary hemorrhage, the Mann–Whitney *U* test and the chi-square test compared the parametric and nonparametric values, respectively. Both univariate and multivariate logistic regression analyzed risk factors for pulmonary hemorrhage. Only variables with statistical significance in univariate analysis were enrolled in multivariate logistic regression analysis. All analyses used SPSS software package, standard version 17.0 (SPSS Inc., Chicago, IL, USA). *P* values were two-sided, and those <0.05 were considered statistically significant.

## 3. Results

### 3.1. Variables Associated with Pulmonary Hemorrhage by Univariate Analysis

Variables related to pulmonary hemorrhage in total 1,100 cases of PCNBs were summarized in Supplementary Table 1. The median (lower quartile to upper quartile) was given here, because all parametric variables showed non-normal distribution. The incidence of pulmonary hemorrhage was 22.4% (246/1100) in total group. Of those, 182 cases (74.0%) were mild (grade 1 in severity of pulmonary hemorrhage). Severe pulmonary hemorrhage (grade 2) occurred in 64 (26.0%) of these 246 cases. There were significant differences between patients with and without pulmonary hemorrhage in small lesion size (*P*=1.210 × 10^−11^), lesion not abutting pleura (*P*=5.431 × 10^−21^), long needle depth to the lesion (*P*=5.653 × 10^−75^), and long dwell time (*P*=1.907 × 10^−8^) (Supplementary Table 1). None of the demographic and diagnostic variables were associated with pulmonary hemorrhage.

### 3.2. Risk Factors for Pulmonary Hemorrhage in Elderly and Young Patients by Univariate Analysis

Risk factors for pulmonary hemorrhage in elderly and young patients were separately evaluated by univariate analysis (Supplementary Table 2 and Supplementary Table 3). The occurrence of pulmonary hemorrhage was 22.1% (96/435) in elderly patients and was 22.6% (150/665) in young patients. In the univariate analysis of the 435 elderly patients studied, patients with pulmonary hemorrhage had significantly smaller lesion size (*P*=6.600 × 10^−5^), lesion not abutting pleura (*P*=1.000 × 10^−6^), longer needle depth to the lesion (*P*=3.123 × 10^−32^), and longer dwell time (*P*=3.350 × 10^−4^) compared to those without pulmonary hemorrhage (Supplementary Table 2). In the univariate analysis of the 665 young patients studied, pulmonary bleeding was significantly influenced by lesion size, lesion abutting pleura, needle depth to the lesion, and dwell time; *P* values were 1.021 × 10^−8^, 3.298 × 10^−16^, 3.304 × 10^−45^, and 1.200 × 10^−5^, respectively (Supplementary Table 3).

### 3.3. Pulmonary Function Parameters Associated with Pulmonary Hemorrhage by Univariate Analysis

Among the patients with pulmonary function spirometries, 25.4% (87/343) patients showed pulmonary hemorrhage. Univariate logistic regression analyzed several pulmonary function parameters for pulmonary hemorrhage (Table 1) including FVC (% pred) (*P*=0.272), FEV_1_ (% pred) (*P*=0.380), and FEV_1_/FVC ratio (%) (*P*=0.593). No previous studies have reported on the correlation between the pulmonary hemorrhage and the small airway function parameters, such as FEF_50%_, FEF_75%_, and FEF_25–75%_, and the large airway function parameters, such as MVV, PEF, and FEF_25%_. However, none of these parameters were associated with the risk of pulmonary hemorrhage.

Furthermore, these 343 patients with pulmonary function results were classified into five groups: patients with normal pulmonary function, patients with obstructive pulmonary dysfunctions, patients with restrictive pulmonary dysfunctions, patients with mixed pulmonary dysfunctions, and patients with small airway dysfunctions (Table 2). However, the incidence of pulmonary hemorrhage was not associated with different types of pulmonary dysfunctions.

The severity of obstructive, restrictive, and mixed dysfunctions was divided into five groups based on the FEV_1_% pred as shown in Table 2. Similarly, the risk of pulmonary hemorrhage did not increase with the severity of pulmonary dysfunctions (*P*=0.560) (Table 2).

### 3.4. Risk Factors of Pulmonary Function Parameters for Pulmonary Hemorrhage in Elderly Patients by Univariate Analysis

Among the 343 patients with pulmonary function results, 128 (37.3%) patients were older than 65 years. On univariate analysis in elderly patients, no significant associations were found between hemorrhage occurrence and pulmonary function parameters (Table 3).

### 3.5. Multivariable Logistic Regression Analysis for Predictors of Pulmonary Hemorrhage in All Patients and in Elderly Patients

Variables with statistical significance in univariate analysis were further enrolled in multivariate logistic regression. Lesion size, needle depth to the lesion, and dwell time were strong predictors of pulmonary hemorrhage in all patients (Table 4). In elderly patients, pulmonary hemorrhage was significantly influenced by needle depth to the lesion and dwell time, while in young patients, pulmonary hemorrhage was independently associated with lesion size, needle depth to the lesion, and dwell time. However, pulmonary function parameters were not independent risk factors for hemorrhage.

## 4. Discussion

The most common manifestation of pulmonary hemorrhage is perilesional ground-glass opacification or new consolidation surrounding the puncture needle track on post-biopsy CT scans, rather than hemoptysis [14]. In our study, the incidence of pulmonary hemorrhage was 22.4%, which was in accordance with other reports [9, 15]. However, César et al. reported lower incidence (15.7%, 37/235) of pulmonary hemorrhage [16]. We believe that inconsistencies in pulmonary hemorrhage rates may be related to performer's experience and sample bias.

Usually, the majority of pulmonary hemorrhage was mild and self-limiting, and only conservative treatments were needed. The patients were instructed to lie in lateral decubitus position, with the puncture side down, keep breathing calmly, and abstain from talking and coughing; when necessary, they were given thrombin and other hemostatic drugs.

Previous studies [9, 10, 14, 17–20] demonstrated that the incidence of pulmonary hemorrhage following lung biopsy was influenced by older age, female sex, lesion location, emphysema, pulmonary hypertension, coaxial technique, and subsolid lesions. With the advancement of techniques, most of the above factors were no longer risk factors. There is no significant difference in the incidence of pulmonary hemorrhage between patients with lung lesions in the lower or upper site. Emphysema, with loss of pulmonary elasticity and irreversible enlargement of the alveoli, was a well-known risk factor for PCNB-induced pneumothorax, but it did not increase the risk of pulmonary bleeding. Here, significant risk factors for pulmonary hemorrhage obtained by multiple logistic regression were lesion size, needle depth to the lesion, and dwell time. The needle depth to the lesion was found to be the most important predictor of hemorrhage, which is consistent with other ﬁndings [9, 17, 21–23]. The occurrence of hemorrhage was significantly higher in patients with a needle trace more than 2.0–2.5 cm [9, 17, 21, 22]. Interestingly, smoking history (pack-years) was a significant risk factor for pneumothorax [24] but not pulmonary hemorrhage. Lesion abutting pleura was a protective factor against pulmonary hemorrhage but was a significant risk factor of pneumothorax [25]. In both elderly patients and young patients, lesions adjacent to the pleura were less prone to pulmonary hemorrhage, but they were not an independent factor.

Hemothorax was extremely rare, with a reported incidence of 0.1% [9]. Hemothorax was caused by the lesion close to the chest wall, and the intercostal puncture damaged the intercostal blood vessels. In this study, no hemothorax was observed. Careful observation of enhanced CT scan before biopsy could avert hemorrhage caused by larger vessels [2]. Our daily operation routine requires intensive preoperative CT examination before all the punctures. If needed, real-time multiplanar reconstruction is performed. These procedures have effectively avoided damage to larger blood vessels and greatly reduced the risk of sever hemorrhage.

As the population ages, and as medical advances make many treatments no longer taboos for the elderly, an increasing proportion of the elderly are receiving PCNB. A primary concern for clinicians is whether abnormal lung function in elderly patients increases the risk of PCNB. It is difficult for doctors to judge which elderly patients should be dissuaded, which patients need to be admitted to the hospital for close observation, and which patients are allowed to go home after a routine 4-hour observation. Emanuela et al. [26] reported that hemorrhage was more common in elderly patients (elderly vs. young: 31 vs. 10%), whereas pneumothorax was more common in young patients (young vs. elderly: 30 vs. 17%). We analyzed the influence of age on the complications in detail. The occurrence of pulmonary hemorrhage was 22.1% in elderly patients and was 22.6% in young patients (*P*=0.668). This is in agreement with several previous studies [1, 9, 19, 27]. Age is no longer a risk factor in evaluating both hemorrhage and pneumothorax [25]. Furthermore, risk factors of pulmonary hemorrhage in different age groups were almost the same here. Smaller lesion size, lesion not abutting pleura, longer needle depth to the lesion, and longer dwell time were significantly higher risk factors of hemorrhage in both elderly patients and young patients. Significant risk factors for pulmonary hemorrhage obtained by multiple logistic regression were needle depth to the lesion and dwell time in elderly patients. In young patients, lesion size should also be considered, because larger lesions were detected in elderly patients (median, lower quartile to upper quartile: 35.4 mm, 24.0–52.0 mm) compared with younger patients (28.0 mm, 18.4–44.4 mm).

The risk factors in pulmonary function parameters for the development of pulmonary hemorrhage were also discussed. Predictive guidance could be concluded from this study in order to take preventive and protective measures for pulmonary dysfunctions. However, whether it was obstructive, restrictive, mixed, or small airway pulmonary dysfunction, the possibility of pulmonary hemorrhage did not increase. The pathological features corresponding to obstructive pulmonary dysfunctions are mainly manifested as chronic bronchitis and emphysema. The changes under the microscope show that the lungs are overexpanded and the elasticity decreases. Thinner alveolar wall, enlarged alveolar cavity, reduced blood supply, and destroyed elastic fiber network could also be detected. It is assumed that patients with abnormal pulmonary dysfunctions are prone to pulmonary hemorrhage, and related mechanisms may include increased capillary permeability, tortuous small blood vessels, high vascular pressure, more small aneurysms, and arteriovenous fistulas. However, in this study, among the 73 patients with obstructive pulmonary dysfunctions, only 26.0% had pulmonary hemorrhage, which was not significantly higher than the incidence of the normal patients (*P*=0.789). Furthermore, the risk of pulmonary hemorrhage did not increase with the severity of pulmonary dysfunctions—that is, patients with severe pulmonary dysfunctions did not have an increased risk of pulmonary hemorrhage in this study. In addition, no pulmonary function parameters were identified as independent predictors for hemorrhage here.

## 5. Conclusion

In this study, age is no longer a risk factor in evaluating pulmonary hemorrhage. Longer needle depth to the lesion and longer dwell time were significantly higher risks of hemorrhage in both elderly patients and young patients. Patients with severe pulmonary dysfunctions did not have an increased risk of pulmonary hemorrhage here.

## Figures and Tables

**Table 1:** Pulmonary function parameters associated with pulmonary hemorrhage evaluated by univariate analysis.

Clinical variables	343 Patients studied		Pulmonary hemorrhage		*Z*	*P* value^†^
	Median	Lower-upper quartile	Yes (*n* = 87)^*∗*^	No (*n* = 256)^*∗*^		
FVC (L)	3.20	2.71–3.84	3.07 (2.54–3.84)	3.24 (2.83–3.85)	−1.735	0.083
FVC (% pred)	103.32	91.97–115.53	107.40 (94.95–115.53)	102.69 (91.39–115.49)	−1.098	0.272
FEV_1_ (L)	2.44	1.95–2.93	2.31 (1.91–2.94)	2.50 (1.96–2.93)	−1.281	0.200
FEV_1_ (% pred)	98.02	81.73–109.90	99.47 (80.62–110.39)	97.42 (81.79–109.69)	−0.877	0.380
FEV_1_/FVC ratio (%)	75.95	69.13–81.21	76.83 (69.40–80.90)	75.86 (69.02–81.29)	−0.534	0.593
FEV1/FVC ratio (% pred)	96.95	89.35–103.23	98.79 (90.64–103.24)	96.57 (88.90–103.17)	−0.731	0.465
FEF_25%_ (% pred)	88.36	63.99–106.12	89.14 (62.37–101.11)	87.87 (64.09–106.69)	−0.536	0.592
FEF_50%_ (% pred)	72.77	48.85–94.61	70.66 (49.71–94.33)	74.48 (48.01–94.94)	−0.110	0.912
FEF_75%_ (% pred)	66.40	44.66–89.90	65.05 (43.40–87.80)	67.85 (45.54–90.71)	−0.579	0.563
FEF_25–75%_ (% pred)	58.02	37.44–78.57	58.35 (35.88–75.36)	57.86 (37.72–79.73)	−0.413	0.680
PEF (% pred)	104.35	84.40–119.59	101.06 (84.13–116.70)	104.72 (85.82–120.01)	−0.850	0.395
MVV (% pred)	95.65	83.28–111.90	94.63 (82.20–109.39)	96.39 (83.34–112.90)	−0.902	0.367

^*∗*^Data are shown as median (lower-upper quartile) for all the numerical values with non-normal distribution. ^†^Mann–Whitney *U* test.

**Table 2:** Relationship between incidence of pulmonary hemorrhage and different types and severity of pulmonary dysfunctions.

Pulmonary function test	Pulmonary hemorrhage		*X* ^*2*^	*P* value^†^
	Yes^*∗*^	No^*∗*^		
Type classification^δ^				
Normal pulmonary function	41	127		
Mixed pulmonary dysfunction	4	18	0.417	0.519
Restrictive pulmonary dysfunction	3	8	0.000	1.000
Obstructive pulmonary dysfunction	19	54	0.072	0.789
Small airway pulmonary dysfunction	20	49	0.537	0.464

Severity^†^	26	80	3.037	0.560
Mild spirometric dysfunction (70 ≤ FEV_1_% pred)	15	39		
Moderate spirometric dysfunction (60 ≤ FEV_1_% pred < 70)	3	20		
Moderately severe spirometric dysfunction (50 ≤ FEV_1_% pred < 60)	3	9		
Severe spirometric dysfunction (35 ≤ FEV_1_% pred < 50)	4	7		
Very severe spirometric dysfunction (FEV_1_% pred < 35)	1	5		

^*∗*^Values are presented as numbers. ^δ^Each group compared with normal ventilation function group. ^⸸^Overall comparison among different severity levels in obstructive, restrictive, and mixed function dysfunctions. ^†^Chi-square test. % pred: percent predicted.

**Table 3:** Pulmonary function parameters in elderly patients associated with pulmonary hemorrhage evaluated by univariate analysis.

Clinical variables	128 elderly patients		Pulmonary hemorrhage		*Z*	*P* value^†^
	Median	Lower-upper quartile	Yes (*n* = 34)^*∗*^	No (*n* = 94)^*∗*^		
FVC (L)	2.99	2.52–3.45	2.67 (2.43–3.49)	3.13 (2.58–3.46)	−1.238	0.216
FVC (% pred)	102.66	87.94–116.35	109.29 (95.44–118.34)	101.84 (85.78–113.99)	−1.602	0.109
FEV_1_ (L)	2.19	1.76–2.64	1.99 (1.68–2.54)	2.29 (1.79–2.64)	−1.168	0.243
FEV_1_ (% pred)	99.51	75.49–110.65	102.08 (75.21–114.83)	98.90 (75.56–109.95)	−0.906	0.365
FEV_1_/FVC ratio (%)	74.19	67.33–80.10	74.40 (67.50–79.40)	73.96 (66.97–80.20)	−0.696	0.486
FEV1/FVC ratio (% pred)	98.49	90.09–107.35	97.41 (90.32–105.36)	98.93 (89.67–107.50)	−0.669	0.504
FEF_25%_ (% pred)	83.34	55.23–104.18	82.68 (57.22–100.47)	83.75 (53.91–106.10)	−0.351	0.726
FEF_50%_ (% pred)	66.95	41.97–92.20	66.43 (40.90–87.30)	67.05 (44.35–93.15)	−0.448	0.654
FEF_75%_ (% pred)	67.85	40.24–91.21	63.64 (38.80–88.73)	70.48 (42.91–91.31)	−0.874	0.382
FEF_25–75%_ (% pred)	54.08	33.14–74.50	50.62 (29.13–74.14)	54.37 (33.25–76.07)	−0.890	0.373
PEF (% pred)	90.78	75.51–111.92	89.07 (69.18–111.03)	92.48 (75.58–112.35)	−0.475	0.635
MVV	92.91	77.22–109.95	92.90 (72.72–109.21)	92.91 (77.61–110.50)	−0.623	0.533

^*∗*^Data are shown as median (lower-upper quartile) for all the numerical values with non-normal distribution. ^†^Mann–Whitney *U* test.

**Table 4:** Multivariable logistic regression model for predictors of pulmonary hemorrhage.

Variable	Odds ratio	95% CI	*P* value
All patients (*n* = 1100)			
Lesion size (mm)	0.989	0.980–0.998	0.012
Lesion abutting pleura	0.784	0.534–1.150	0.214
Needle depth to the lesion (mm)	1.111	1.093–1.129	4.361 × 10^−36^
Dwell time (min)	1.239	1.141–1.346	3.727 × 10^−7^

Elder patients (*n* = 435)			
Lesion size (mm)	0.997	0.987–1.006	0.496
Lesion abutting pleura	0.866	0.470–1.594	0.643
Needle depth to the lesion (mm)	1.128	1.097–1.161	5.482 × 10^−17^
Dwell time (min)	1.251	1.107–1.415	3.400 × 10^−4^

Young patients (*n* = 665)			
Lesion size (mm)	0.975	0.959–0.991	0.002
Lesion abutting pleura	0.749	0.455–1.233	0.256
Needle depth to the lesion (mm)	1.104	1.082–1.128	7.925 × 10^−21^
Dwell time (min)	1.226	1.093–1.374	4.950 × 10^−4^

Bold values mean that significant difference exists.

## Data Availability

All data generated and analyzed during the current study are included in this published article.

## Abbreviations:

CT: Computed tomography
PCNB: Percutaneous core needle biopsy
FNA: Fine-needle aspiration
PT: Prothrombin
APTT: Activated partial thromboplastin time
FVC: Forced vital capacity
FEV_1_: Forced expiratory volume in one second
% pred: Percent predicted
FEF_X%_: Instantaneous forced expiratory flow when X% of the FVC has been expired
FEF_25–75%_: Mean forced expiratory flow between 25% and 75% of FVC
PEF: Peak expiratory flow
MVV: Maximum voluntary ventilation.

## Supplementary Materials

Supplementary Materials Supplementary Table 1: variables of total PCNBs with and without pulmonary hemorrhage. Supplementary Table 2: variables of elderly patients with and without pulmonary hemorrhage. Supplementary Table 3: variables of young patients with and without pulmonary hemorrhage.
