# Supplementary material for: Older Age and Abnormal Pulmonary Ventilation Function Do Not Increase the Risk of Pulmonary Hemorrhage Caused by CT-Guided Percutaneous Core Needle Biopsy
Source: Can Respir J. 2022 Aug 5;2022:5238177. doi: 10.1155/2022/5238177 (PMC9410978; doi:10.1155/2022/5238177)
Supplement: Supplementary Materials — Supplementary Table 1: variables of total PCNBs with and without pulmonary hemorrhage. Supplementary Table 2: variables of elderly patients with and without pulmonary hemorrhage. Supplementary Table 3: variables of young patients with and without pulmonary hemorrhage. [file 5238177.f1.zip › 5238177.f1/Supplementary Table 1.docx]

**Supplementary Table 1 Variables of total PCNBs with and without pulmonary hemorrhage**

|  | **Total PCNBs (*n* =1100)*** | **Pulmonary Hemorrhage** | | | | | |
| --- | --- | --- | --- | --- | --- | --- | --- |
|  | ****N* (%) or**  **Median (lower-upper quartile** | **Yes (*n* = 246)**  ****N* (%) or**  **Median (lower-upper quartile** | **No (*n* = 854)**  ****N* (%) or**  **Median (lower-upper quartile** | ***Z/X^2^*** | | | ***P* Value^†^** |
| **Demographic variables** |  |  |  | |  |  | |
| Age (years) | 62.0 (53.0–68.0) | 61.0 (53.0–68.0) | 62.0 (53.0–69.0) | | -0.429 | 0.668 | |
| Sex |  |  |  | | 2.127 | 0.145 | |
| Male | 648 (58.9%) | 135 (20.8%) | 513 (79.2%) | |  |  | |
| Female | 452 (41.1%) | 111 (24.6%) | 341 (75.4%) | |  |  | |
| Smoking history (pack-years) | 0.0 (0.0–23.0) | 0.0 (0.0–20.0) | 0.0 (0.0–27.0) | | -1.379 | 0.168 | |
| Prior thoracic surgery |  |  |  | | - | 1.000 | |
| Yes | 4 (0.4%) | 1 (25.0%) | 3 (75.0%) | |  |  | |
| No | 1096 (99.6%) | 245 (22.4%) | 851 (77.6%) | |  |  | |
| Prior thoracic radiotherapy |  |  |  | | 0.061 | 0.806 | |
| Yes | 8 (0.7%) | 1 (12.5%) | 7 (87.5%) | |  |  | |
| No | 1092 (99.3%) | 245 (22.4%) | 847 (77.6%) | |  |  | |
| Prior chemotherapy |  |  |  | | 0.008 | 0.928 | |
| Yes | 15 (1.4%) | 4 (26.7%) | 11 (73.3%) | |  |  | |
| No | 1085 (98.6%) | 242 (22.3%) | 843 (77.7%) | |  |  | |
| **Lesion variables** |  |  |  | |  |  | |
| Lesion site |  |  |  | | 1.863 | 0.172 | |
| Upper | 629 (57.2%) | 150 (23.8%) | 479 (76.2%) | |  |  | |
| Lower | 471 (42.8%) | 96 (20.4%) | 375 (79.6%) | |  |  | |
| Lesion size (mm) | 30.6 (20.8–47.9) | 25.3 (17.3–34.1) | 33.8 (21.9–52.0) | | -6.779 | **1.210×10^-11^** | |
| Lesion abutting pleura |  |  |  | | 88.369 | **5.431×10^-21^** | |
| Yes | 738 (67.1%) | 104 (14.1%) | 634 (85.9%) | |  |  | |
| No | 362 (32.9%) | 142 (39.2%) | 220 (60.8%) | |  |  | |
| Emphysema along the needle path |  |  |  | | 0.751 | 0.386 | |
| Yes | 80 (7.3%) | 21 (26.2%) | 59 (73.8%) | |  |  | |
| No | 1020 (92.7%) | 225 (22.1%) | 795 (77.9%) | |  |  | |
| **Technique variables** |  |  |  | |  |  | |
| Patient position |  |  |  | | 4.812 | 0.090 | |
| Supine | 387 (35.2%) | 81 (20.9%) | 306 (79.1%) | |  |  | |
| Prone | 639 (58.1%) | 141 (22.1%) | 498 (77.9%) | |  |  | |
| Lateral decubitus | 74 (6.7%) | 24 (32.4%) | 50 (67.6%) | |  |  | |
| Needle puncture site |  |  |  | | 6.822 | 0.146 | |
| Anterior | 181 (16.5%) | 40 (22.1%) | 141 (77.9%) | |  |  | |
| Anterolateral | 177 (16.1%) | 37 (20.9%) | 140 (79.1%) | |  |  | |
| Lateral | 115 (10.5%) | 35 (30.4%) | 80 (69.6%) | |  |  | |
| Posterior | 517 (47. 0%) | 116 (22.4%) | 401 (77.6%) | |  |  | |
| Posterolateral | 110 (10.0%) | 18 (16.4%) | 92 (83.6%) | |  |  | |
| Needle depth to the lesion (mm) | 0.0 (0.0–16.0) | 19.80 (13.4–28.0) | 0.0 (0.0–10.0) | | -18.321 | **5.653×10^-75^** | |
| Dwell time (min) | 4.3 (3.5–5.0) | 4.7 (3.9–5.2) | 4.3 (3.3–5.0) | | -5.620 | **1.907×10^-8^** | |
| Needle-pleural angle (º) | 63.0 (50.0–80.0) | 62.0 (50.0–78.2) | 65.0 (50.0–80.0) | | -1.051 | 0.293 | |
| Needle redirection |  |  |  | | 2.016 | 0.156 | |
| Yes | 34 (3.1%) | 11 (32.4%) | 23 (67.6%) | |  |  | |
| No | 1066 (96.9%) | 235 (22.0%) | 831 (78.0%) | |  |  | |
| **Diagnostic variables** |  |  |  | | 1.355 | 0.736 | |
| Malignant | 755 (68.6%) | 176 (23.3%) | 579 (76.7%) | |  |  | |
| Benign | 310 (28.2%) | 63 (20.3%) | 247 (79.7%) | |  |  | |
| Borderline | 2 (0.2%) | 0 (0.0%) | 2 (100.0%) | |  |  | |
| Non-Diagnostic / Inadequate | 33 (3.0%) | 7 (21.2%) | 26 (78.8%) | |  |  | |

* Data are shown as number *N* (%) for categorical variables or median (lower quartile to upper quartile) for quantitative variables with non-normal distribution.

^⸹^ Range only for quantitative variables.

^†^ Chi-square test for categorical variables. Mann-Whitney U test for quantitative variables. All quantitative data showed non-normal distribution by Shapiro-Wilk test.
