# Supplementary material for: Older Age and Abnormal Pulmonary Ventilation Function Do Not Increase the Risk of Pulmonary Hemorrhage Caused by CT-Guided Percutaneous Core Needle Biopsy
Source: Can Respir J. 2022 Aug 5;2022:5238177. doi: 10.1155/2022/5238177 (PMC9410978; doi:10.1155/2022/5238177)
Supplement: Supplementary Materials — Supplementary Table 1: variables of total PCNBs with and without pulmonary hemorrhage. Supplementary Table 2: variables of elderly patients with and without pulmonary hemorrhage. Supplementary Table 3: variables of young patients with and without pulmonary hemorrhage. [file 5238177.f1.zip › 5238177.f1/Supplementary Table 2.docx]

**Supplementary Table 2 Variables of elderly patients with and without pulmonary hemorrhage**

|  | **Elderly patients (*n* =435)***  **Median (lower-upper quartile)** | **Pulmonary Hemorrhage** | | ***X^2^/Z*** | ***P* Value^†^** |
| --- | --- | --- | --- | --- | --- |
|  |  | **Yes (*n* = 96)*** | **No (*n* = 339)*** |  |  |
| **Demographic variables** |  |  |  |  |  |
| Age (years) | 70.0 (67.0–74.0) | 70.00 (67.0–74.8) | 70.0 (67.0–74.0) | -0.143 | 0.886 |
| Sex |  |  |  | 0.455 | 0.500 |
| Male | 280 (64.4%) | 59 (21.1%) | 221 (78.9%) |  |  |
| Female | 155 (35.6%) | 37 (23.9%) | 118 (76.1%) |  |  |
| Smoking history (pack-years) | 0.0 (0.0–40.0) | 0.0 (0.0–30.0) | 0.0 (0.0–40.0) | -0.617 | 0.537 |
| Prior thoracic surgery |  |  |  | - | 0.393 |
| Yes | 2 (0.5%) | 1 (50.0%) | 1 (50.0%) |  |  |
| No | 433 (99.5%) | 95 (21.9%) | 338 (78.1%) |  |  |
| Prior thoracic radiotherapy |  |  |  | **–** | 1.000 |
| Yes | 3 (0.7%) | 0 (0.0%) | 3 (100.0%) |  |  |
| No | 432 (99.3%) | 96 (22.2%) | 336 (77.8%) |  |  |
| Prior chemotherapy |  |  |  | 0.185 | 0.667 |
| Yes | 5 (1.1%) | 2 (40.0%) | 3 (60.0%) |  |  |
| No | 430 (98.9%) | 94 (21.9%) | 336 (78.1%) |  |  |
| **Lesion variables** |  |  |  |  |  |
| Lesion site |  |  |  | 0.801 | 0.371 |
| Upper | 250 (57.5%) | 59 (23.6%) | 191 (76.4%) |  |  |
| Lower | 185 (42.5%) | 37 (20.0%) | 148 (80.0%) |  |  |
| Lesion size (mm) | 35.4 (24.0–52.0) | 27.5 (22.3–39.0) | 37.7 (25.0–54.1) | -3.988 | **6.600×10^-5^** |
| Lesion abutting pleura |  |  |  | 23.370 | **1.000×10^-6^** |
| Yes | 323 (74.3%) | 53 (16.4%) | 270 (83.6%) |  |  |
| No | 112 (25.7%) | 43 (38.4%) | 69 (61.6%) |  |  |
| Emphysema along the needle path |  |  |  | 0.221 | 0.638 |
| Yes | 32 (7.4%) | 6 (18.8%) | 26 (81.3%) |  |  |
| No | 403 (92.6%) | 90 (22.3%) | 313 (77.7%) |  |  |
| **Technique variables** |  |  |  |  |  |
| Patient position |  |  |  | 2.507 | 0.286 |
| Supine | 157 (36.1%) | 36 (22.9%) | 121 (77.1%) |  |  |
| Prone | 251 (57.7%) | 51 (20.3%) | 200 (79.7%) |  |  |
| Lateral decubitus | 27 (6.2%) | 9 (33.3%) | 18 (66.7%) |  |  |
| Needle puncture site |  |  |  | 7.567 | 0.109 |
| Anterior | 73 (16.8%) | 20 (27.4%) | 53 (72.6%) |  |  |
| Anterolateral | 61 (14.0%) | 12 (19.7%) | 49 (80.3%) |  |  |
| Lateral | 54 (12.4%) | 18 (33.3%) | 36 (66.7%) |  |  |
| Posterior | 200 (46.0%) | 39 (19.5%) | 161 (80.5%) |  |  |
| Posterolateral | 47 (10.8%) | 7 (14.9%) | 40 (85.1%) |  |  |
| Needle depth to the lesion (mm) | 0.0 (0.0–14.0) | 18.2 (12.0–25.2) | 0.0 (0.0–7.3) | -11.819 | **3.123×10^-32^** |
| Dwell time (min) | 4.3 (3.5–5.0) | 4.7 (4.0–5.3) | 4.3 (3.5–5.0) | -3.587 | **3.350×10^-4^** |
| Needle-pleural angle (º) | 65.0 (50.0–82.0) | 60.0 (50.0–78.0) | 65.7 (51.0–82.0) | -1.538 | 0.124 |
| Needle redirection |  |  |  | 0.000 | 1.000 |
| Yes | 12 (2.8%) | 3 (25.0%) | 9 (75.0%) |  |  |
| No | 423 (97.2%) | 93 (22.0%) | 330 (78.0%) |  |  |
| **Diagnostic variables** |  |  |  | 0.378 | 0.842 |
| Malignant | 329 (75.6%) | 75 (22.8%) | 254 (77.2%) |  |  |
| Benign | 100 (23.0%) | 20 (20.0%) | 80 (80.0%) |  |  |
| Borderline | 0 (0.0%) | 0 (0.0%) | 0 (0.0%) |  |  |
| Non-Diagnostic/inadequate | 6 (1.4%) | 1 (16.7%) | 5 (83.3%) |  |  |

* Data are shown as number *N* (%) for categorical values or median (lower quartile to upper quartile) for numerical values with non-normal distribution.

^†^ Chi-square test for categorical values. Kruskal-Wallis H test for quantitative values. All quantitative values showed non-normal distribution by Shapiro-Wilk test.
