# Supplementary material for: Older Age and Abnormal Pulmonary Ventilation Function Do Not Increase the Risk of Pulmonary Hemorrhage Caused by CT-Guided Percutaneous Core Needle Biopsy
Source: Can Respir J. 2022 Aug 5;2022:5238177. doi: 10.1155/2022/5238177 (PMC9410978; doi:10.1155/2022/5238177)
Supplement: Supplementary Materials — Supplementary Table 1: variables of total PCNBs with and without pulmonary hemorrhage. Supplementary Table 2: variables of elderly patients with and without pulmonary hemorrhage. Supplementary Table 3: variables of young patients with and without pulmonary hemorrhage. [file 5238177.f1.zip › 5238177.f1/Supplementary Table 3.docx]

**Supplementary Table 3 Variables of young patients with and without pulmonary hemorrhage**

|  | **Young patients (*n* =665)***  **Median (lower-upper quartile)** | **Pulmonary Hemorrhage** | | | | ***X^2^/Z*** | ***P* Value^†^** | | |
| --- | --- | --- | --- | --- | --- | --- | --- | --- | --- |
|  |  | **Yes (*n* = 150)*** | **No (*n* = 515)*** | | |  |  |  |  |
| **Demographic variables** |  |  |  | | |  |  | | |
| Age (years) | 55.0 (49.0–61.0) | 54.0 (49.0–60.0) | 55.0 (49.0–61.0) | | | -0.495 | 0.621 | | |
| Sex |  |  |  | | | 1.710 | 0.191 | | |
| Male | 368 (55.3%) | 76 (20.7%) | 292 (79.3%) | | |  |  | | |
| Female | 297 (44.7%) | 74 (24.9%) | 223 (75.1%) | | |  |  | | |
| Smoking history (pack-years) | 0.0 (0.0–13.0) | 0.0 (0.0–2.0) | 0.0 (0.0–15.0) | | | -1.264 | 0.206 | | |
| Prior thoracic surgery |  |  |  | | | - | 1.000 | | |
| Yes | 2 (0.3%) | 0 (0%) | 2 (100.0%) | | |  |  | | |
| No | 663 (99.7%) | 150 (22.6%) | 513 (77.4%) | | |  |  | | |
| Prior thoracic radiotherapy |  |  |  | | | 0.000 | 1.000 | | |
| Yes | 5 (0.8%) | 1 (20.0%) | 4 (80.0%) | | |  |  | | |
| No | 660 (99.2%) | 149 (22.8%) | 511 (77.4%) | | |  |  | | |
| Prior chemotherapy |  |  |  | | | 0.000 | 1.000 | | |
| Yes | 10 (1.5%) | 2 (20.0%) | 8 (80.0%) | | |  |  | | |
| No | 655 (98.5%) | 148 (22.6%) | 507 (77.4%) | | |  |  | | |
| **Lesion variables** |  |  |  | | |  |  | | |
| Lesion site |  |  |  | | | 1.067 | 0.302 | | |
| Upper | 379 (57.0%) | 91 (24.0%) | 288 (76.0%) | | |  |  | | |
| Lower | 286 (43.0%) | 59 (20.6%) | 227 (79.4%) | | |  |  | | |
| Lesion size (mm) | 28.0 (18.4–44.4) | 23.0 (15.8–32.3) | 30.1 (20.0–48.5) | | | -5.727 | **1.021×10^-8^** | | |
| Lesion abutting pleura |  |  |  | | | 66.616 | **3.298×10^-16^** | | |
| Yes | 415 (62.4%) | 51 (12.3%) | 364 (87.7%) | | |  |  | | |
| No | 250 (37.6%) | 99 (39.6%) | 151 (60.4%) | | |  |  | | |
| Emphysema along the needle path |  |  |  | | | 2.238 | 0.135 | | |
| Yes | 48 (7.2%) | 15 (31.2%) | 33 (68.8%) | | |  |  | | |
| No | 617 (92.8%) | 135 (21.9%) | 482 (78.1%) | | |  |  | | |
| **Technique variables** | |  |  |  |  | | |  |  |
| Patient position |  |  |  | | | 3.625 | 0.163 | | |
| Supine | 230 (34.6%) | 45 (19.6%) | 185 (80.4%) | | |  |  | | |
| Prone | 388 (58.3%) | 90 (23.2%) | 298 (76.8%) | | |  |  | | |
| Lateral decubitus | 47 (7.1%) | 15 (31.9%) | 32 (68.1%) | | |  |  | | |
| Needle puncture site |  |  |  | | | 3.543 | 0.471 | | |
| Anterior | 108 (16.2%) | 20 (18.5%) | 88 (81.5%) | | |  |  | | |
| Anterolateral | 116 (17.4%) | 25 (21.6%) | 91 (78.4%) | | |  |  | | |
| Lateral | 61 (9.2%) | 17 (27.9%) | 44 (72.1%) | | |  |  | | |
| Posterior | 317 (47.7%) | 77 (24.3%) | 240 (75.7%) | | |  |  | | |
| Posterolateral | 63 (9.5%) | 11 (17.5%) | 52 (82.5%) | | |  |  | | |
| Needle depth to the lesion (mm) | 5.5 (0.0–17.1) | 20.0 (13.9–29.4) | 0.0 (0.0–11.0) | | | -14.110 | **3.304×10^-45^** | | |
| Dwell time (min) | 4.3 (3.3–5.0) | 4.7 (3.8–5.0) | 4.3 (3.3–5.0) | | | -4.374 | **1.200×10^-5^** | | |
| Needle-pleural angle (º) | 63.0 (50.0–79.0) | 64.0 (50.0–78.5) | 63.0 (50.0–79.6) | | | -0.001 | 0.999 | | |
| Needle redirection |  |  |  | | | 1.733 | 0.188 | | |
| Yes | 22 (3.3%) | 8 (36.4%) | 14 (63.6%) | | |  |  | | |
| No | 643 (96.7%) | 142 (22.1%) | 501 (77.9%) | | |  |  | | |
| **Diagnostic variables** |  |  |  | | | 1.078 | 0.819 | | |
| Malignant | 426 (64.1%) | 101 (23.7%) | 325 (76.3%) | | |  |  | | |
| Benign | 210 (31.6%) | 43 (20.5%) | 167 (79.5%) | | |  |  | | |
| Borderline | 2 (0.3%) | 0 (0.0%) | 2 (100.0%) | | |  |  | | |
| Non-Diagnostic / Inadequate | 27 (4.1%) | 6 (22.2%) | 21 (77.8%) | | |  |  | | |

* Data are shown as number *N* (%) for categorical values or median (lower quartile to upper quartile) for numerical values with non-normal distribution.

^†^ Chi-square test for categorical values. Kruskal-Wallis H test for quantitative values. All quantitative values showed non-normal distribution by Shapiro-Wilk test.
